# Drug–drug interactions between sucroferric oxyhydroxide and losartan, furosemide, omeprazole, digoxin and warfarin in healthy subjects

**DOI:** 10.1007/s40620-014-0080-1

**Published:** 2014-04-04

**Authors:** Edward Chong, Veena Kalia, Sandra Willsie, Peter Winkle

**Affiliations:** 1 Vifor Pharma, Clinical Development, Aspreva International Ltd., 1203, 4464 Markham Street, Victoria, BC V8Z 7X8, Canada; 2 PRA International, Lenexa, KS USA; 3 Anaheim Clinical Trials, Anaheim, CA USA

**Keywords:** Chronic kidney disease, Hyperphosphatemia, Sucroferric oxyhydroxide, Pharmacokinetics, Phase I

## Abstract

**Background:**

The novel iron-based phosphate binder sucroferric oxyhydroxide is being investigated for the treatment of hyperphosphatemia. Patients with chronic kidney disease often have multiple comorbidities that may necessitate the daily use of several types of medication. Therefore, the potential pharmacokinetic drug–drug interactions between sucroferric oxyhydroxide and selected drugs commonly taken by dialysis patients were investigated.

**Methods:**

Five Phase I, single-center, open-label, randomized, three-period crossover studies in healthy volunteers investigated the effect of a single dose of sucroferric oxyhydroxide 1 g (based on iron content) on the pharmacokinetics of losartan 100 mg, furosemide 40 mg, omeprazole 40 mg, digoxin 0.5 mg and warfarin 10 mg. Pharmacokinetic parameters [including area under the plasma concentration–time curve (AUC) from time 0 extrapolated to infinite time (AUC_0–∞_) and from 0 to 24 h (AUC_0–24_)] for these drugs were determined: alone in the presence of food; with sucroferric oxyhydroxide in the presence of food; 2 h after food and sucroferric oxyhydroxide administration.

**Results:**

Systemic exposure based on AUC_0–∞_ for all drugs, and AUC_0–24_ for all drugs except omeprazole (for which AUC 0–8 h was measured), was unaffected to a clinically significant extent by the presence of sucroferric oxyhydroxide, irrespective of whether sucroferric oxyhydroxide was administered with the drug or 2 h earlier.

**Conclusions:**

There is a low risk of drug–drug interactions between sucroferric oxyhydroxide and losartan, furosemide, digoxin and warfarin. There is also a low risk of drug–drug interaction with omeprazole (based on AUC_0–∞_ values). Therefore, sucroferric oxyhydroxide may be administered concomitantly without the need to adjust the dosage regimens of these drugs.

## Introduction

One of the serious and common clinical consequences of chronic kidney disease (CKD) is hyperphosphatemia, which is associated with CKD-mineral bone disorder [1], and increased risk of cardiovascular events [2] and mortality [3–6]. As a result, patients on dialysis often require phosphate binding agents to control serum phosphorus concentrations.

The novel polynuclear iron (III)-oxyhydroxide phosphate binder sucroferric oxyhydroxide is being investigated for the treatment of hyperphosphatemia. It is formulated as a chewable tablet that may be taken without water. In Phase I clinical studies, sucroferric oxyhydroxide was shown to be associated with minimal iron absorption through the gastrointestinal (GI) tract, and to be well tolerated [7, 8]. A Phase II dose-finding study demonstrated that sucroferric oxyhydroxide doses of 1.0–2.5 g/day (based on iron content) significantly lowered serum phosphorus concentrations [9]. A Phase III study in patients with hyperphosphatemia undergoing hemo- or peritoneal dialysis was recently undertaken [10]. In this study, patients (n = 1,059) were randomized to receive sucroferric oxyhydroxide 1.0–3.0 g/day or sevelamer 4.8–14.4 g/day. It was shown that sucroferric oxyhydroxide was non-inferior to sevelamer in terms of serum phosphorus control over the first 12 weeks of treatment, maintained its phosphorus-lowering effect over 52 weeks with a lesser pill burden than sevelamer, and was associated with reduced non-adherence to treatment [10, 11].

Patients with CKD often have multiple comorbidities, including cardiovascular disease, hypertension and diabetes, which may necessitate the use of several different types of daily medications [12, 13]. In vitro studies have identified a few potential interactions between sucroferric oxyhydroxide and some common medications (unpublished data) prescribed to patients with CKD. Therefore, a program of human in vivo pharmacokinetic (PK) drug–drug interaction (DDI) studies between sucroferric oxyhydroxide and several common medications was undertaken. Here we report data from five separate Phase I clinical DDI studies undertaken in healthy adults of sucroferric oxyhydroxide administered with medications selected based on adsorption of these medications onto sucroferric oxyhydroxide from in vitro investigations. Digoxin and warfarin did not show interactions in the in vitro investigations, but were chosen due to their narrow therapeutic window. The primary objective of these studies was to assess whether there was any effect of sucroferric oxyhydroxide on medication exposure [area under the plasma concentration–time curve from time 0–24 h (AUC_0–24_); AUC from time 0 extrapolated to infinite time (AUC_0–∞_); peak serum concentration (C_max_); time to C_max_ (T_max_) and terminal half-life (t_1/2_)]. Adverse event profiles and routine biochemical/hematological laboratory tests were also assessed.

## Subjects and methods

### Study design and interventions

Five Phase I, single-center, open-label, randomized, three-period crossover studies investigated the PK effect of sucroferric oxyhydroxide (single dose of 1 g, based on iron content) on the following medications: losartan potassium (Cozaar^®^ 100 mg), furosemide (Lasix^®^ 40 mg), omeprazole (Prilosec^®^ 40 mg), digoxin (Lanoxin^®^ 0.5 mg) and warfarin (Coumadin^®^ 10 mg). The doses of these drugs are based on the approved doses and those that are commonly used in clinical practice and/or have been used in PK interaction studies. The dose of sucroferric oxyhydroxide is the maximum single dose proposed for clinical use. Each study comprised a screening visit, 12 safety and PK visits, two washout periods (7 days each) and one follow-up visit 2 weeks after the last administration of study medication.

Subjects were randomized (1:1:1) to one of three treatment groups by sequentially following a randomization code list. Treatment Group 1 began with ‘Schedule 1’ dosing, Treatment Group 2 began with ‘Schedule 2’ dosing, and Treatment Group 3 began with ‘Schedule 3’ dosing (Table 1). After a 7-day washout period, subjects from Treatment Group 1 crossed over to receive ‘Schedule 2’ dosing, Treatment Group 2 crossed over to receive ‘Schedule 3’ dosing, and Treatment Group 3 crossed over to receive ‘Schedule 1’ dosing. After a further 7-day washout period, subjects from Treatment Group 1 crossed over to receive ‘Schedule 3’ dosing, Treatment Group 2 crossed over to receive ‘Schedule 1’ dosing, and Treatment Group 3 crossed over to receive ‘Schedule 2’ dosing.

**Table 1:** Study drug administration

Dosing schedule	Day −1	Day 1	Day 8^a^/11	Day 9^a^/12	Day 16^a^/22	Day 17^a^/23
1	Sucroferric oxyhydroxide TID^b^	Sucroferric oxyhydroxide single dose^c^ Test drug^d^		Test drug^d^	Sucroferric oxyhydroxide TID^b^	Sucroferric oxyhydroxide single dose^c^ Test drug^e^
2		Test drug^d^	Sucroferric oxyhydroxide TID^b^	Sucroferric oxyhydroxide single dose^c^ Test drug^e^	Sucroferric oxyhydroxide TID^b^	Sucroferric oxyhydroxide single dose^c^ Test drug^d^
3	Sucroferric oxyhydroxide TID^b^	Sucroferric oxyhydroxide single dose^c^ Test drug^e^	Sucroferric oxyhydroxide TID^b^	Sucroferric oxyhydroxide single dose^c^ Test drug^d^		Test drug^d^

^a^Warfarin interaction study only

^b^Sucroferric oxyhydroxide 1 g (based on iron content) three times daily (TID; 6 tablets/day; total daily dose of 3 g/day, based on iron content) given with meals

^c^Sucroferric oxyhydroxide 1 g (based on iron content) given as a single dose of two tablets with breakfast

^d^Test drug single dose given with breakfast

^e^Test drug single dose given 2 h after breakfast/sucroferric oxyhydroxide administration but ≥1 h before the next meal

### Participants

Subjects eligible for these studies were healthy male or female volunteers aged 20–50 years, and with a body mass index of 18–32 kg/m^2^. The subjects had to provide written informed consent before the commencement of any study-specific procedures. All protocols were approved by an independent Review Board. Subjects were ineligible for the studies if they had participated in a clinical trial with an investigational drug or device ≤3 months before screening, if they had a history of clinically significant disorders or drug hypersensitivity, had a history of recurrent infectious diseases or major illness ≤30 days before screening, used nicotine ≤30 days before Study Day −1, presented with clinically significant abnormal findings on any screening assessments, took any medication ≤2 weeks before Study Day −1, had a clinically relevant history of drug or alcohol misuse, suffered any significant blood loss or blood donation ≤3 months before Study Day −1, or if they were pregnant or did not use adequate contraceptive precautions. No non-study medications, nicotine, alcohol or drugs of abuse were permitted during the study.

### Outcomes

The primary endpoints of the studies were AUC_0–24_, AUC_0–∞_, C_max_, T_max_, and t_1/2_ for each of the study medications. These endpoints were analyzed for the two active enantiomers of warfarin (R- and S-forms) and also for the active metabolite of losartan (EXP 3174). Safety endpoints included adverse events and routine biochemical/hematological laboratory tests.

### Sample sizes

For the studies involving losartan and furosemide, 36 and 42 subjects, respectively, were considered to be adequate in terms of demonstrating bioequivalence for the primary analysis of AUC_0–24_, and the sample sizes were not based on statistical assumptions.

For the omeprazole and digoxin studies, a total of 36 evaluable subjects each was judged to be sufficient to demonstrate the equivalence in a crossover design with a power of 84 %, considering a standard deviation (SD) of the difference of 0.43 (in the log scale), a 1-sided *α* of 0.05, an expected ratio of 1, and the bioequivalence limits of 80–125 %.

For the warfarin study, at least 36 evaluable subjects were considered sufficient to demonstrate the bioequivalence in a crossover design with a power of more than 95 %, considering a SD of the difference of 0.35 (in the log scale), a 1-sided *α* of 0.05, an expected ratio of 1, and the bioequivalence limits of 80–125 %.

### Statistical methods

PK parameters were calculated using non-compartmental methods with WinNonlin^®^ Professional Version 5.1.1 or higher (Pharsight Corp., Mountain View, CA, USA). PK computations were performed in SAS^®^ Version 9.1 or higher. The plasma PK parameters were estimated from the concentration–time profiles for all PK population subjects. In estimating the PK parameters, values that were below the lower limit of quantification (LLOQ) at the beginning of the profile were set to 0. Lower limit of quantification values that occurred after the first quantifiable point were considered missing. Values that were embedded between LLOQs, or quantifiable values occurring after two or more LLOQs, were set to missing at the discretion of the pharmacokineticist. Descriptive statistics were used to summarize the calculated PK parameters by treatment. Missing PK parameter data were not imputed. T_max_ and t_1/2_ were analyzed using the nonparametric Wilcoxon signed-rank test. To assess the effect of sucroferric oxyhydroxide on the PK of test medications (or the active metabolite), an analysis of variance (ANOVA) with fixed treatment, period, sequence, and subject within sequence was applied to natural logarithm-transformed AUC_0–24_, AUC_0–∞_, and C_max_. For omeprazole, analysis of AUC_0–24_ was planned but drug levels were below the limit of quantitation in all subjects by 8–12 h. Therefore, AUC 0–8 h (AUC_0–8_) was calculated instead of AUC_0–24_.

Bioequivalence criteria for log-transformed parameters were defined as 80–125 %. No clinically significant differences were concluded when 90 % confidence intervals (CIs) for exposure ratios fell within these bioequivalence criteria.

## Results

### Subject demographics and disposition

In total, 213 subjects were randomized across the five studies, of whom 210 received study medication and were included in the safety population. Overall, 200 subjects received treatment in at least two of the three dosing schedules and were eligible for PK analysis (n = 36 for losartan, n = 41 for furosemide, n = 39 for omeprazole, n = 42 for digoxin, and n = 42 for warfarin).

In total, 193 subjects completed the studies and 17 withdrew. Of the subjects who withdrew before randomization, nine did so by their own decision, six were found to have taken drugs/alcohol; following randomization, one subject was withdrawn as a result of an adverse event (rhabdomyolysis), and a second subject was withdrawn as the result of an administrative decision.

Patient demographics at baseline were similar between the treatment groups (Table 2).

**Table 2:** Summary of demographics for randomized subjects across the five Phase I studies

Demographic characteristics	Losartan (n = 41)	Furosemide (n = 41)	Omeprazole (n = 43)	Digoxin (n = 42)	Warfarin (n = 43)
Sex, n (%)					
Male	26 (63.4)	28 (68.3)	22 (51.2)	21 (50.0)	26 (60.5)
Female	15 (36.6)	13 (31.7)	21 (48.8)	21 (50.0)	17 (39.5)
Race, n (%)					
White	31 (75.6)	26 (63.4)	30 (69.8)	27 (64.3)	26 (60.5)
Black/African American	3 (7.3)	5 (12.2)	5 (11.6)	10 (23.9)	15 (34.9)
Asian	7 (17.1)	10 (24.4)	7 (16.3)	4 (9.5)	1 (2.3)
Other	0 (0.0)	0 (0.0)	1 (2.3)	1 (2.4)	1 (2.3)
Ethnicity, n (%)					
Not Hispanic or Latino	24 (58.5)	28 (68.3)	26 (60.5)	24 (57.1)	41 (95.3)
Hispanic or Latino	17 (41.5)	13 (31.7)	17 (39.5)	18 (42.9)	2 (4.7)
Age, mean (SD) years	31.8 (9.5)	31.8 (8.5)	31.4 (9.6)	31.5 (8.6)	30.1 (7.8)

*SD* standard deviation

### Pharmacokinetic results

#### AUC_0–24_ and AUC_0–∞_

The systemic exposure of all test medications was unaffected to a clinically significant extent by the presence of sucroferric oxyhydroxide (i.e., 90 % CIs were within the bioequivalence range), based on AUC_0–∞_ (Table 3). Moreover, the systemic exposure of medications was unaffected irrespective of whether sucroferric oxyhydroxide was administered with the medication or 2 h earlier.

**Table 3:** Effect of sucroferric oxyhydroxide on test drug pharmacokinetic exposure (geometric LS means), based on AUC_0–24_, AUC_0–∞_ and C_max_

PK parameter (units)	No sucroferric oxyhydroxide; test drug with food (Schedule 2)	Sucroferric oxyhydroxide and test drug with food (Schedule 1)	Sucroferric oxyhydroxide with food; test drug 2 h later (Schedule 3)	Geometric LS mean ratio (90 % CI); Schedule 1/Schedule 2 Schedule 3/Schedule 2
Losartan				
AUC_0–24_ (h ng/ml)^a^	782.00	773.60	742.27	0.989 (0.927, 1.056) 0.949 (0.889, 1.013)
AUC_0–∞_ (h ng/ml)^a^	786.87	774.71	743.86	0.985 (0.923, 1.050) 0.945 (0.886, 1.009)
C_max_ (ng/ml)	313.90	290.93	382.76	0.927 (0.761, 1.129) 1.219 (1.000, 1.487)
EXP 3174				
AUC_0–24_ (h ng/ml)^a^	4,104.09	3,920.49	4,071.18	0.955 (0.857, 1.065) 0.992 (0.889, 1.107)
AUC_0–∞_ (h ng/ml)^a^	4,500.93	4,236.16	4,399.30	0.941 (0.847, 1.046) 0.977 (0.878, 1.088)
C_max_ (ng/ml)	631.26	588.79	659.77	0.933 (0.808, 1.077) 1.045 (0.904, 1.208)
Furosemide				
AUC_0–24_ (h ng/ml)^a^	2,083.94	1,851.81	2,003.20	0.889 (0.844, 0.935) 0.961 (0.913, 1.012)
AUC_0–∞_ (h ng/ml)^a^	2,159.70	2,030.60	2,103.79	0.940 (0.886, 0.998) 0.974 (0.920, 1.031)
C_max_ (ng/ml)	585.22	497.44	682.49	0.850 (0.766, 0.944) 1.166 (1.050, 1.296)
Omeprazole				
AUC_0–8_ (h ng/ml)^a^	1,295.43	1,255.25	1,491.18	0.969 (0.869, 1.080) 1.151 (1.034, 1.282)
AUC_0–∞_ (h ng/ml)^a^	1,620.82	1,479.14	1,592.40	0.913 (0.830, 1.003) 0.982 (0.903, 1.069)
C_max_ (ng/ml)	571.42	495.34	741.80	0.867 (0.754, 0.996) 1.298 (1.130, 1.492)
Digoxin				
AUC_0–24_ (h ng/ml)^a^	11.11	12.11	12.13	1.090 (1.014, 1.171) 1.091 (1.015, 1.173)
AUC_0–∞_ (h ng/ml)^a^	30.55	32.79	31.02	1.074 (0.998, 1.155) 1.016 (0.944, 1.093)
C_max_ (ng/ml)	1.62	1.80	1.98	1.109 (0.995, 1.236) 1.218 (1.093, 1.356)
R-warfarin				
AUC_0–24_ (h ng/ml)^a^	19,061.33	19,049.28	19,726.65	0.999 (0.981, 1.018) 1.035 (1.016, 1.054)
AUC_0–∞_ (h ng/ml)^a^	68,552.25	68,867.47	69,945.84	1.005 (0.975, 1.035) 1.020 (0.991, 1.051)
C_max_ (ng/ml)	1,084.79	1,084.43	1,179.19	1.000 (0.970, 1.030) 1.087 (1.055, 1.120)
S-warfarin				
AUC_0–24_ (h ng/ml)^a^	15,836.22	15,801.93	16,202.49	0.998 (0.976, 1.021) 1.023 (1.000, 1.047)
AUC_0–∞_ (h ng/ml)^a^	46,174.66	45,824.90	46,277.24	0.992 (0.960, 1.026) 1.002 (0.969, 1.036)
C_max_ (ng/ml)	1,033.36	1,035.07	1,168.21	1.002 (0.965, 1.040) 1.130 (1.088, 1.174)

*LS* least squares, *PK* pharmacokinetic, *AUC* area under the curve

^a^Although AUC_0–24_ was planned, omeprazole levels were below the limit of quantitation in all subjects by 8–12 h; therefore, AUC_0–8_ was calculated instead of AUC_0–24_

The systemic exposure, based on AUC_0–24_, for all drugs except omeprazole (for which AUC_0–8_ was measured) was also largely unaffected by the presence of sucroferric oxyhydroxide, irrespective of whether sucroferric oxyhydroxide was administered with the medication or 2 h earlier.

The AUC_0–8_ value of omeprazole was increased when sucroferric oxyhydroxide was administered 2 h before the medication, as the upper bound 90 % CI was outside the bioequivalence range, relative to that obtained in the absence of sucroferric oxyhydroxide.

The extent of formation of the active metabolite of losartan, EXP 3174, was also unaffected by sucroferric oxyhydroxide, with AUC parameters being within the bioequivalence range.

#### C_max_

Sucroferric oxyhydroxide had no clinically significant effect on the C_max_ of R-warfarin, S-warfarin or EXP 3174 (i.e., 90 % CIs were within the bioequivalence range) (Table 3). However, the C_max_ values of losartan, furosemide and omeprazole were decreased (lower bound 90 % CIs were outside the bioequivalence range) relative to those in the absence of sucroferric oxyhydroxide.

Furthermore, when sucroferric oxyhydroxide (and food) was administered 2 h before each of the medications, the C_max_ values of losartan, furosemide, omeprazole and digoxin were increased (upper bound 90 % CIs were outside the bioequivalence range) relative to those in the absence of sucroferric oxyhydroxide.

#### T_max_ and t_1/2_

The T_max_ value of losartan was significantly reduced when sucroferric oxyhydroxide was administered with the medication relative to the value obtained in the absence of sucroferric oxyhydroxide (Table 4). The T_max_ values of losartan, EXP 3174, furosemide, omeprazole, digoxin and R- and S-warfarin were also significantly reduced when sucroferric oxyhydroxide (and food) was administered 2 h before the medications.

**Table 4:** Effect of sucroferric oxyhydroxide on test drug pharmacokinetic exposure (median difference), based on T_max_ and t_1/2_

PK parameter (units)	No sucroferric oxyhydroxide; test drug with food (Schedule 2)	Sucroferric oxyhydroxide and test drug with food (Schedule 1)	Sucroferric oxyhydroxide with food; test drug 2 h later (Schedule 3)	Median difference (90 % CI) Schedule 1/Schedule 2 Schedule 3/Schedule 2	Wilcoxon signed rank p value
Losartan					
T_max_ (h)	3.00	2.50	2.00	−1.00 (−1.25, −0.50) −1.00 (−1.50, −0.74)	0.0021 <0.0001
t_1/2_ (h)	2.08	1.83	1.97	−0.25 (−0.46, −0.08) −0.12 (−0.47, 0.13)	0.0154 0.4774
EXP 3174					
T_max_ (h)	5.00	4.52	4.00	−0.50 (−1.00, 0.00) −1.00 (−1.50, −0.50)	0.0810 0.0019
t_1/2_ (h)	6.94	6.55	6.87	−0.35 (−0.63, 0.09) −0.19 (−0.50, 0.13)	0.0415 0.3248
Furosemide					
T_max_ (h)	3.00	3.00	2.00	−0.45 (−0.75, 0.00) −1.25 (−1.72, −0.75)	0.1004 <0.0001
t_1/2_ (h)	2.88	5.77	2.84	2.02 (0.80, 3.23) 0.22 (−1.31, 1.84)	0.0044 0.7003
Omeprazole					
T_max_ (h)	4.00	5.00	2.50	0.50 (0.00, 1.00) −1.75 (−2.25, −1.25)	0.2142 <0.0001
t_1/2_ (h)	1.00	1.09	0.92	0.00 (−0.13, 0.18) −0.01 (−0.11, 0.06)	1.0000 0.6889
Digoxin					
T_max_ (h)	1.50	2.00	1.50	0.02 (0.00, 0.25) −0.50 (−0.50, −0.25)	0.2445 0.0006
t_1/2_ (h)	39.52	38.12	35.93	−2.13 (−5.02, 1.26) −2.80 (−5.32, −0.15)	0.4022 0.0854
R-warfarin					
T_max_ (h)	3.08	4.00	2.00	0.25 (−0.50, 0.75) −1.50 (−2.25, −1.00)	0.7835 <0.0001
t_1/2_ (h)	48.60	48.43	48.31	0.40 (−0.95, 1.83) −0.32 (−1.57, 0.99)	0.5520 0.6775
S-warfarin					
T_max_ (h)	3.00	3.51	1.50	0.25 (−0.25, 0.51) −1.25 (−1.50, −1.00)	0.4823 <0.0001
t_1/2_ (h)	40.10	40.55	40.44	0.30 (−0.32, 0.99) −0.74 (−1.59, 0.30)	0.3817 0.2612

*CI* confidence interval, *PK* pharmacokinetic

A significant decrease in the t_1/2_ of losartan and EXP 3174 and an increase in t_1/2_ of furosemide were observed when sucroferric oxyhydroxide (and food) was administered with the medications, as compared to when medications were administered ìn the absence of sucroferric oxyhydroxide (Table 4).

### Safety

Across the five studies, 126/210 (60.0 %) subjects experienced treatment-emergent adverse events (TEAEs). Overall, 82/210 (39.1 %) subjects reported treatment-related TEAEs. These were reported by an average of 22.9 % (n = 47/205) of subjects during ‘Schedule 1’ dosing, 5.0 % (n = 10/199) during ‘Schedule 2’ dosing, and 22.0 % (n = 44/200) during ‘Schedule 3’ dosing. GI events were the most common class of treatment-related TEAE, occurring in an average of 30.0 % (n = 63/210) of subjects across studies. Of these, discolored feces were the most frequently reported treatment-related GI event and occurred in an average of 20.0 % of subjects across the studies.

One subject in the warfarin study developed a TEAE (rhabdomyolysis) that was considered to be both serious and severe, but unrelated to study treatment. The subject reported an unplanned period of strenuous physical activity a few days prior to complaining of muscle soreness that was associated with elevated muscle enzymes. The event resolved spontaneously within a few days. There were no deaths across the studies.

## Discussion

The data reported here indicate that sucroferric oxyhydroxide does not affect systemic exposure (based on AUC_0–∞_) to any of the drugs tested in this study when they are administered with sucroferric oxyhydroxide or 2 h after sucroferric oxyhydroxide. Similarly, sucroferric oxyhydroxide does not affect systemic exposure (based on AUC_0–24_) for any of the drugs, irrespective of whether sucroferric oxyhydroxide was administered with the medication or 2 h earlier. However, no AUC_0–24_ values could be obtained for omeprazole because drug levels were below the limit of quantification in all subjects by 8–12 h but AUC_0–∞_ values were within the bioequivalence criteria. Taken together, the results indicate that sucroferric oxyhydroxide may be administered concomitantly with losartan, furosemide, omeprazole, digoxin or warfarin without the need to adjust drug dosages or administration regimen.

The study data also suggest that the C_max_ values of losartan, furosemide, omeprazole and digoxin are sensitive to the timing of sucroferric oxyhydroxide administration. When losartan, furosemide, omeprazole and digoxin were administered 2 h after sucroferric oxyhydroxide (and food), but ≥1 h before the next meal, their C_max_ values were increased relative to those observed when the medications were given with food in the absence of sucroferric oxyhydroxide. It is possible that these differences are caused by a ‘food effect’. C_max_ values for furosemide, omeprazole and digoxin have been observed to decrease when these agents are administered with food [14–16]. However, a direct effect of sucroferric oxyhydroxide on the rate of absorption of these compounds cannot be excluded because sucroferric oxyhydroxide was associated with decreased C_max_ values for losartan (although the C_max_ of its active metabolite EXP 3174 was unaffected), furosemide and omeprazole when given with the drugs relative to C_max_ values in the absence of sucroferric oxyhydroxide—food being given with the agents in both cases. A similar action may underlie the observed effects of sucroferric oxyhydroxide on the T_max_ and t_1/2_ of some agents investigated in these studies. However, given that the overall exposure for these agents was largely unaffected by sucroferric oxyhydroxide, as discussed above, these changes are unlikely to be clinically significant.

Sucroferric oxyhydroxide was generally well tolerated when administered with the medications in healthy subjects. The TEAEs related to treatment were almost entirely non-severe and consistent with the known safety profile of sucroferric oxyhydroxide [7, 9, 10]. Discolored feces were a common GI-related event among subjects after receiving sucroferric oxyhydroxide. Discolored feces are a known effect of sucroferric oxyhydroxide as well as other iron-based phosphate binders [9, 17] and iron-based products in general.

In previous clinical studies, sucroferric oxyhydroxide was effective at reducing serum phosphorus concentrations in CKD patients undergoing dialysis, and showed similar efficacy and tolerability to sevelamer [9, 10], while having a lower tablet burden [10]. Therefore, sucroferric oxyhydroxide may represent a new treatment option for CKD dialysis patients, with the potential for improved adherence and low risk of DDIs with the medications investigated in these studies.
